# The Potential Use of *N*-Myristoyltransferase as a Biomarker in the Early Diagnosis of Colon Cancer

**DOI:** 10.3390/cancers3011372

**Published:** 2011-03-16

**Authors:** Sujeet Kumar, Jonathan R Dimmock, Rajendra K Sharma

**Affiliations:** 1 Department of Pathology and Laboratory Medicine, College of Medicine, University of Saskatchewan, Saskatoon, Saskatchewan S7N OW8, Canada; E-Mail: sujeet.kumar@saskcancer.ca; 2 Cancer Research Unit, Saskatchewan Cancer Agency, 20 Campus Drive, Saskatoon, SK S7N 4H4 Canada; 3 Drug Design and Discovery Research Group, College of Pharmacy and Nutrition, University of Saskatchewan, Saskatoon, Saskatchewan S7N 5C9, Canada; E-Mail: jr.dimmock@usask.ca

**Keywords:** colon cancer, human adenocarcinoma, *N*-myristoyltransferase, protein myristoylation

## Abstract

Colon cancer is one of the most common malignant diseases and a major cause of mortality in the Western world. Metastasis to lymph nodes and other gastrointestinal organs, especially to the liver and lungs, is most common and occurs in up to 25% of cancer patients when initially diagnosed. The majority of colon cancers develop from noncancerous adenomatous polyps on the lining of the colon which grow over the years to become cancerous. If detected early, the surgical resections of the growth, often in combination with chemotherapy, significantly increases life expectancy. We have shown that the enzyme *N*-myristoyltransferase (NMT) which carries out lipid modification of several proteins (including many of those involved in oncogenesis) is expressed at higher levels in cancerous tissues from the colon. We have also shown that in peripheral blood mononuclear cells (PBMC) and bone marrow (BM) cells collected from colon cancer patients and from azoxymethane-induced rats the expression and localization of NMT is altered. We have observed strong positivity for NMT in immunohistochemical analysis for PBMC from colon cancer patients as compared to control groups. Furthermore, in the bone marrow (BM) mononuclear cells, NMT was found to be confined to the nuclei whereas in control groups it was observed to be located in the cytoplasm. In conclusion, this strikingly differential localization offers the basis of a potential investigational tool for screening or diagnosis of individuals at risk for or suspected of having colon cancer.

## 1. Introduction

Colorectal cancer is one of the most common forms of malignancy worldwide and is associated with high mortality [1]. It is the second most common cause of cancer associated deaths in the Western world [2,3] and is the fourth most common cause of malignancy in the United States [4,5]. Colon cancer generally develops from polyps on the lining of the colon which ultimately becomes cancerous, although not in all cases [6]. It is one of the most curable forms of cancer if detected early. The Tumor-Node-Metastasis (TNM) system is the primary prognostic approach to identify the differences among patients with the early stages of this disease [4,5]. However, among patients with similar pathological stages, the survival outcomes are not similar. The identification of molecular markers of more aggressive colorectal cancers have been in increasing demand in order to tailor patient therapy or to identify the disease well in advance.

Lipidic modification of proteins has received great attention recently as targets for therapeutic interventions to cancer [7-10]. One such candidate is the N-myristoylation process catalyzed by the enzyme N-myristoyltransferase [9,10]. The protein belongs to the GNAT superfamily of enzymes [11] and carries out irreversible lipidic modifications; the covalent attachment of myristate, a 14 carbon saturated fatty acid, generally to the N-terminal glycine residue of proteins [9,10,12-15]. Myrisitic acid is considered a rare fatty acid in the cells and constitutes less than 1% of the total fatty acid pool [12]. However it is estimated that at least approximately 0.5% of eukaryotic proteins are myristoylated [9,15]. This suggests a special role for myristoylation which cannot be substituted by other lipidic modifications of proteins.

Many of the myristoylated proteins are involved in signaling cascades, cellular transformation and oncogenesis [12,13]. The myristoylation requirement of the viral oncogene pp60v-src for membrane association and cell transformation was the very first suggestion of the importance of protein myristoylation in tumorigenesis [16-18]. Myristoylated proteins have been shown to play a role in colon cancer progression. Myristoylated tyrosine kinases pp60^c-src^ and pp60^c-yes^ are several fold higher in colonic pre-neoplastic lesions and neoplasms than normal colon cells [19-21]. The elevated NMT activity during colonic carcinogenesis may be due to the higher demand for myristoylation of various proteins/oncoproteins which are overexpressed and activated during tumorogenesis. A direct relationship between elevated NMT expression and activity in colon cancer progression has been reported [22-23]. A differential expression of pp60^c-src^ has been observed in colonic tumor-derived cell lines [19,21] and colonic polyps prone to developing cancer [24]. It has also been observed that in colon cancer cell lines elevated expression of NMT correlates with high levels of c-Src levels [25]. Higher levels of cytoskeletal-associated pp60^c-src^ protein tyrosine kinase activity have been observed in intestinal crypt cells along with higher expression of pp60^c-yes^ in the normal intestinal epithelium [26-27]. Studies have revealed that pp60^c-src^ is overexpressed in human colon carcinoma and it has enhanced kinase activity in progressive stages and metastases of human colorectal cancer [19-20]. Recent studies show that src kinase activity is positively regulated by myristoylation and the non-myristoylated c-Src has reduced kinase activity [28]. Blockage of N-myristoylation in colonic cell lines has been shown to compromise colony formation and proliferation and also show reduced localization of pp60^c-src^ to the plasma membrane [29]. The studies suggest that NMT represents both a valuable clinical marker and a therapeutic target for cancer [30-32]. There are several other putative biological markers that play important roles in the pathogenesis, proliferation and invasion of colonic tumors. Examples include epidermal growth factor receptor (EGFR), c-MET, β-catenin and p53 [33]. However, a limitation of assessing the expression of these markers or measuring NMT expression and activity for prognostic/diagnostic purposes is that an invasive biopsy must be performed in order to obtain tumor tissue for protein analysis [22-23,33].

Development of quick and rapid blood-based diagnostic biomarkers for the early detection of colorectal tumors will greatly facilitate the screening or diagnosis of individuals at risk of, or suspected of having, colon cancer. The current review summarizes the developments in using NMT as a suitable sensitive and specific biomarker which merits further investigation to perform a risk assessment for the early stages of colorectal neoplasia.

## 2. Elevated NMT Activity in Colonic Tumors

NMT has been established as a clinical candidate for diagnosing colorectal cancer after the observation that the enzyme is significantly elevated in colorectal tumors. Significantly, high NMT activity has been observed in tumor tissue samples from the azoxymethane-induced rat model for colonic tumors as well as from human patients [22,23]. The azoxymethane-induced colon cancer rat model has been used extensively to characterize changes in various enzymes during colon carcinogenesis [34] and produces tumors which are histologically similar to human colonic neoplasms and follow the adenocarcinoma sequence [35]. Induction of tumor growth in the rat model was done by subcutaneous injection of azoxymethane over a specified period of time (eight weeks) [22]. The colon from the rats was obtained 28 weeks after the induction period and the number of colonic tumors in each rat ranged from one to twelve [22]. The histological evaluation of these tumors showed that they ranged from adenoma (polyp) to highly invasive C2 tumors, based on the modified Dukes' staging system [36]. A total of 35 colonic tumors were analyzed from 10 rats [22]. Normal-appearing mucosa (at least 1 cm from any tumor; scraped free from the underlying muscle layer) was obtained from seven of the 10 tumor-bearing rats (three rats did not show normal-appearing mucosa that was at least 1 cm from any tumor to allow analysis). Normal colonic mucosa obtained from three control rats was also used in the study [22].

Analysis of the resulting 45 rat colonic tissue specimens showed that NMT activity is higher in colonic tumors than the normal and normal-appearing colonic mucosa. Elevations in NMT activity are reported in human colon adenocarcinoma as compared to normal appearing mucosa or normal mucosa [22]. An analysis of the tumor sub-types based on the histological evaluation of the tumors showed that elevated NMT activity in colon tumor tissues reveals that adenomas (polyps) and tumors of stage Bl have the highest elevated NMT activity [22]. Furthermore, other diseases of the colon (Crohn's disease and volvulus colon) did not reflect any elevations in NMT activity suggesting that elevated NMT activity does not non-specifically occur in inflammatory conditions or in non-cancerous lesions [22]. The marked elevation in rat adenomatous polyps and stage Bl tumors suggests that NMT activity is elevated in the early stages of colonic carcinogenesis [22]. However, the lack of an available NMT antibody at the time of these studies precluded the determination of whether this effect is due to a higher specific activity of the enzyme or because of higher expression levels of the enzyme in neoplastic tissues.

## 3. Human Colorectal Adenocarcinoma and NMT Expression Levels

**Figure 1.**
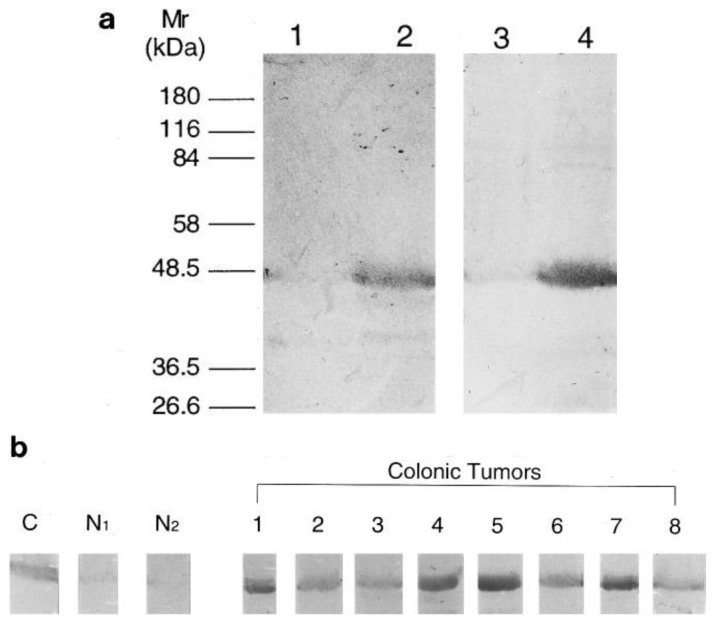
(**a**) Equal amounts of protein extracts from normal mucosa (lanes 1 and 3) and colorectal tumor tissue (lanes 2 and 4) immunoblotted with anti-hNMT (lanes 1 and 2) and anti-peptide NMT (lanes 3 and 4) antibodies. (**b**) Immunoblot of equal amount of protein extracts from control NMT, normal mucosa, and colorectal tumor tissue extracts with anti-peptide NMT antibodies; c, control recombinant human NMT; N1 and N2, normal mucosa; [1-8], colonic tumor samples (Adapted from [23]).

The recombinant expression of human NMT in *E. coli* [37] greatly facilitated the expression and purification of NMT for antibody generation for subsequent studies. Analysis of human colorectal adenocarcinoma following NMT antibody development showed that NMT expression is higher in colorectal tumor tissues [23]. With the use of anti-human NMT antibody and antibodies specific to the *N*-terminal amino acid residues 97-112 of the human NMT [23], it was found that NMT in both normal mucosa and colorectal tumor tissue specimens are of a molecular mass close to 48.5 kDa (Figure 1a). However, the immunoblot analysis showed that the NMT levels are elevated only in tumor tissue samples (Figure 1b).

**Figure 2.**
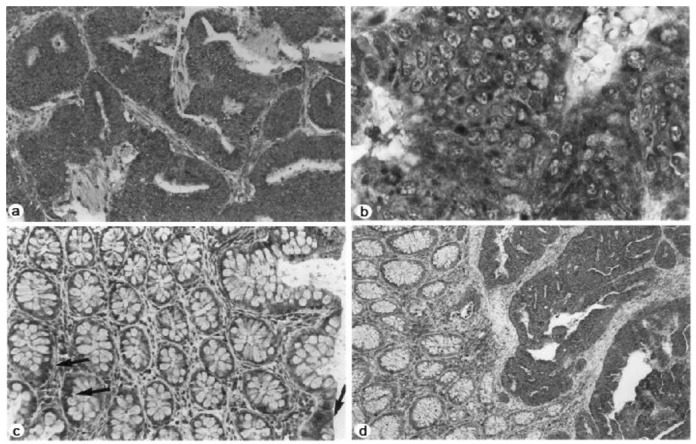
(**a**) Section from colorectal adenocarcinoma showing a marked degree of antibody reactivity in most of the tumor cells (immunoperoxidase; original magnification, 120×). (**b**) High-power view depicting the cytoplasmic staining of the tumor cells. The nuclei appeared as ovoid spaces delimited by a nuclear membrane (immunoperoxidase; original magnification, 600×). (**c**) Section of normal mucosa far removed from tumor showing a mild degree of focal staining (see arrows) (immunoperoxidase; original magnification, 120×). (**d**) Section with transitional mucosa (on the left) showing a mild to moderate degree of diffuse reactivity compared to the strong tumor reactivity on the right (immunoperoxidase; original magnification, 120×) (Adapted from Ref. [23]).

Furthermore, the immunohistochemical studies showed increased staining for NMT in colorectal tumors compared to normal mucosa. More than 50% of the cells appeared to be positive in immunohistochemical analysis with anti-peptide antibody in all eight cases of human adenocarcinoma tissue samples studied (Figure 2a). The staining predominantly appeared to be cytoplasmic rather than nuclear (Figure 2b). The mucosal sections distant from the tumor site showed mild reactivity (Figure 2c); however the transitional mucosa in the vicinity of the cancers stained more than normal mucosa albeit not to the same degree as the tumors (Figure 2d).

## 4. NMT as a Marker for Early Diagnosis of Colon Cancer

**Figure 3.**
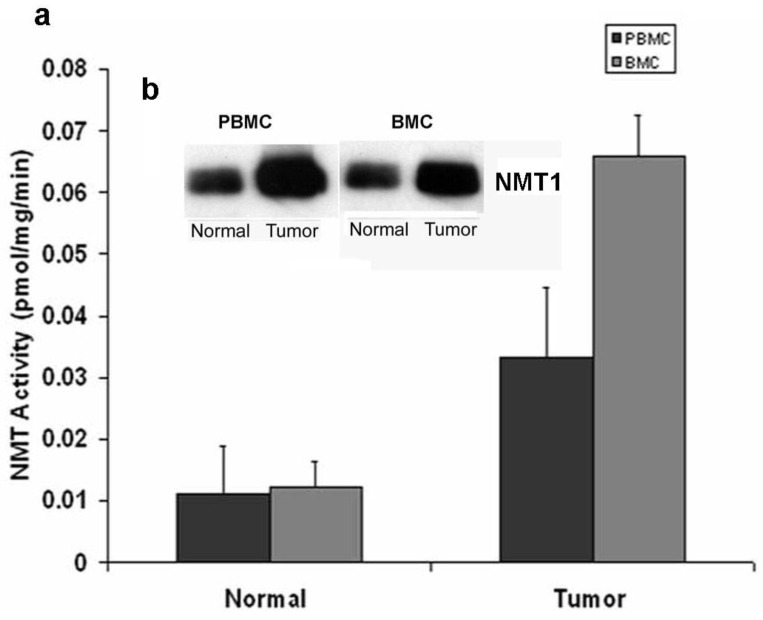
NMT activity in peripheral blood mononuclear cells (PBMC) and bone marrow (BM) cells of normal and colorectal tumor bearing rats. (**a**) Isolated PMBC from peripheral blood of control or tumor bearing rat assessed for NMT activity using cAMP-dependent protein kinase derived peptide substrate. Values are mean ± SD of three independent experiments. (**b**) Western blot analysis of equal amounts of protein extracts from PMBC and BM cells of normal and colorectal tumor bearing rats (probed with monoclonal anti-human NMT antibody (1:250 dilutions)) (Adapted from ref. [32]).

Marked elevations in NMT activity in adenomatous polyps and stage B1 tumors in azoxymethane-induced rat models of colon cancer and the increased NMT expression levels establish it as a target molecule for early screening of colorectal cancer [22,23]. To establish a rapid detection system which eliminates invasive biopsy to obtain tumor tissues from the colon, NMT activity and expression profiles were studied in peripheral blood mononuclear cells (PBMC) and bone marrow (BM) cells of azoxymethane-induced rat models for colon tumors [32]. A strikingly differential expression profile and significantly higher activity has been observed in tumor bearing rats (n = 20) as compared to control rats (n = 10). NMT activity in tumor bearing rats was three-fold higher in PBMC whereas it was elevated by five-fold in BM cells as compared to control group (Figure 3a).

**Figure 4.**
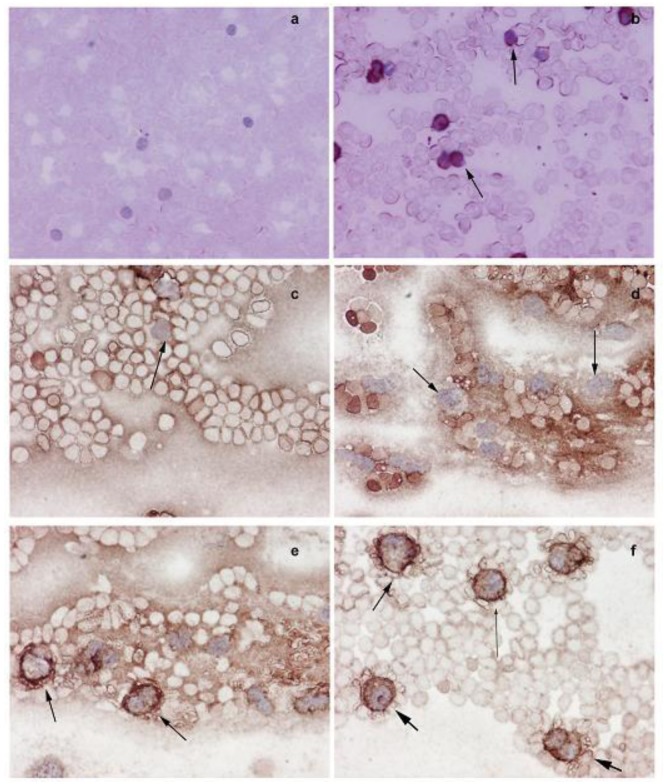
Immunohistochemical analysis of peripheral blood mononuclear cells (PBMC) of normal and tumor bearing hosts. Smears of peripheral blood cells were incubated with anti-NMT antibody. (**a**) PBMC (mostly lymphocytes) from control rats devoid of NMT staining, (**b**) intense NMT expression observed in the PBMC of colorectal tumor bearing rats as evident from strong staining (see arrows), (**c**) negative staining of lymphocytes (see arrows), (**d**) negative staining of monocytes (see arrows) in peripheral blood smear of control, (**e**) peripheral blood smear of colon cancer patients show positive staining of macrophages (arrows), (**f**) peripheral blood smear of colon cancer patients show positive staining of neutrophil (fat arrows), lymphocyte (lean long arrow) and macrophages (arrow) (Adapted from Ref. [32]).

The Western blot analysis shows that the increased NMT activity in PBMC and BM cells is concurrent with elevated levels of NMT expression (Figure 3b). The immunohistochemical analysis of peripheral blood smear for NMT shows very rare or no positivity in a control group (Figure 4a) whereas moderate-strong staining of more than 50% mononuclear cells is observed in samples from tumor-bearing rats (Figure 4b). In PBMC of rats bearing highly invasive tumors, intense NMT expression was observed [32]. Further immunohistochemical studies on human PBMC from colon cancer patients showed that NMT staining in the mononuclear cells (including lymphocytes and monocytes) and neutrophils in the peripheral blood smears of the healthy controls ranged from negative to rare weak positivity (Figure 4c and 4d). The positive staining for NMT in PBMC's of control subjects is less than 20%. However, strong NMT staining in more than 80% of the cells was observed in monocytes, lymphocytes and neutrophils in the blood smear of the colon cancer patient (Figure 4e and 4f).

**Figure 5.**
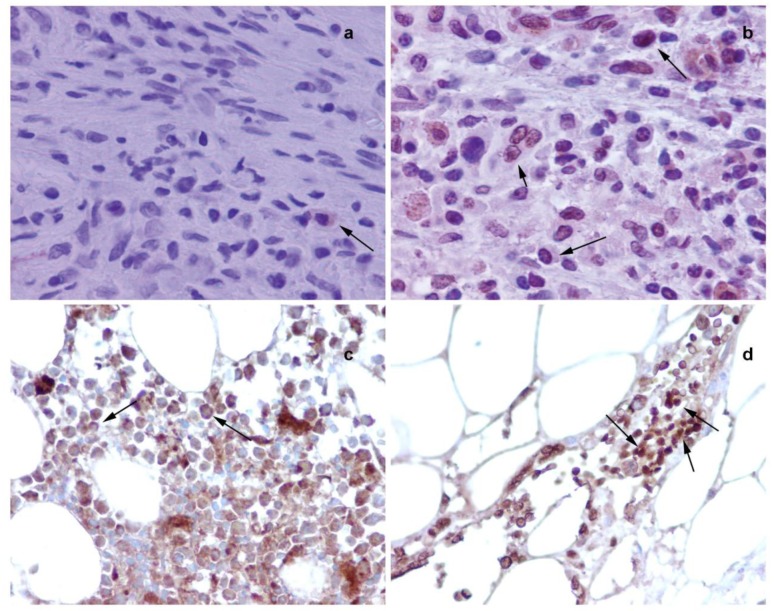
Immunohistochemical analysis of bone marrow of normal and tumor bearing hosts. (**a**) Cytoplasmic staining of NMT (see arrow) in bone marrow cells from control rats, (**b**) nuclear localization of NMT in bone marrow cells from tumor bearing rat (see arrows), (**c**) mostly cytoplasmic NMT staining in bone marrow of control (see arrows) and (**d**) intense nuclear (and some cytoplasmic) staining for NMT observed in the bone marrow of colon cancer patient (see arrows) (Adapted from Ref. [32]).

Further immunohistochemical analysis on BM sections of colon tumor bearing rats (Figure 5a and 5b) and colon cancer patients (Figure 5c and 5d) have shown that in tumor bearing rats and colon cancer patients, NMT is localized in the nuclei as well as in the cytoplasm of the bone marrow mononuclear cells (Figure 5b and 5d) whereas in the control bone marrow specimens it remains cytoplasmic (Figure 5a and 5c). The differential NMT expression offers the basis of a potential adjunct investigative tool for screening or diagnosis of patients at risk of, or suspected of having, colon cancer. The altered localization of NMT in the BM of tumor bearing hosts may serve as an added investigative tool for diagnostic purposes. The findings are suggestive that NMT is a potential novel marker for the diagnosis of colon cancer.

## 5. Summary

There has been a rapid increase in the incidence of cancer in the last two decades and cancer mortality continues to grow day by day. About 27 million new cases of cancer and 17.5 million deaths due to cancer are expected by 2050 [38]. It is a prevalent disease in this modern era and the elevation of cancer incidences has imposed an ever-mounting economic burden to health care systems. Despite various treatment modalities that include chemotherapy, radiation therapy, surgery, gene therapy, transfusion, transplantation, laser and heat therapy, the treatment of cancer remains elusive and early detection is the key to effective cure. Colon cancer contributes a major fraction of cancer associated deaths worldwide and the mortality rates could be significantly reduced if the disease was detected at early stages. Detection of elevated NMT levels in the blood provides NMT as a potential promising simple diagnostic biomarker for early detection of colorectal tumors. A follow up study with larger samples size is underway for its validation and to establish NMT levels as a blood based marker for the early diagnosis of colon cancer.
